# Cytogenetic evidence and *dmrt* linkage indicate male heterogamety in a non-bilaterian animal

**DOI:** 10.1371/journal.pone.0285851

**Published:** 2023-05-18

**Authors:** Joshua Vacarizas, Takahiro Taguchi, Takuma Mezaki, Sam Edward Manalili, Rei Kawakami, Satoshi Kubota

**Affiliations:** 1 Kuroshio Science Program, Graduate School of Integrated Arts and Sciences, Kochi University, Nankoku, Kochi, Japan; 2 Department of Nutrition, Faculty of Health Science, Kochi Gakuen University, Asahitenjin-Cho, Kochi, Japan; 3 Kuroshio Biological Research Foundation, Otsuki, Hata County, Kochi, Japan; 4 Agriculture and Marine Science Program, Graduate School of Integrated Arts and Sciences, Kochi University, Nankoku, Kochi, Japan; 5 Kuroshio Science Unit, Multidisciplinary Science Cluster, Kochi University, Nankoku, Kochi, Japan; Laboratoire de Biologie du Développement de Villefranche-sur-Mer, FRANCE

## Abstract

The diversity of sex determination systems in animals suggests that sex chromosomes evolve independently across different lineages. However, the present data on these systems is largely limited and represented mainly by bilaterian animals. Sex chromosomes and sex determination system based on cytogenetic evidence remain a mystery among non-bilaterians, the most basal animals. Here, we investigated the sex determination system of a non-bilaterian (*Goniopora djiboutiensis*) based on karyotypic analysis and identification of locus of *dmrt1*, a known master sex-determining gene in many animals. Results showed that among the three isolated *dmrt* genes, *GddmrtC* was sperm-linked. Fluorescence in situ hybridization revealed that 47% of the observed metaphase cells contained the *GddmrtC* locus on the shorter chromosome of the heteromorphic pair, whereas the other 53% contained no *GddmrtC* locus and pairing of the longer chromosome of the heteromorphic pair was observed. These findings provided the cytogenetic evidence for the existence of the Y sex chromosome in a non-bilaterian animal and supports male heterogamety as previously reported in other non-bilaterian species using RAD sequencing. The Y chromosome-specific *GddmrtC* sequence was most homologous to the vertebrate *dmrt1*, which is known for its role in male sex determination and differentiation. Our result on identification of putative sex chromosomes for *G*. *djiboutiensis* may contribute into understanding of the possible genetic sex determination systems in non-bilaterian animals.

## Introduction

Sex of animals is determined by either environmental sex determination (ESD), genetic sex determination (GSD), or both. ESD is exhibited primarily by some reptiles, fish, and certain species of invertebrates (crustaceans, worms, hydrozoans), in which the sex of the animal is dictated by temperature or other environmental cues. GSD, on the other hand, is the most widely recognized sex determination mechanism in animals. In GSD, sex is generally determined by the presence of a sex chromosome that carries the key genes responsible for the development of male or female-specific characteristics. Various GSD systems based on different sex chromosome configurations have been reported in animals. These are male heterogamety (XX/XY) in mammals and many insects; female heterogamety (ZZ/ZW) in birds, reptiles, and Lepidoptera insects; homomorphic sex chromosomes in some reptiles; and haplodiploidy in some arthropods [1–3]. The diversity and complexity of these sex determination systems appear to have no clear evolutionary patterns, which formed the consensus understanding that sex chromosomes evolve independently across different lineages [4, 5]. However, current empirical data on sex determination systems of animals are still highly limited and biased towards certain groups, as previous investigations are limited among the bilaterians. The GSD system from a non-bilaterian animal might provide important insight into the sex determination mechanisms of basal animals, which may contribute to the overall understanding of the evolution of sex determination systems and sex chromosomes in animals.

The conventional approach to identify the GSD system of an organism is based on cytogenetic methods. Using cytogenetic data, chromosome structures and organization are revealed in karyotypes, from which sex chromosomes can be identified. This method serves as the foundation for the discovery of various sex determination systems among many important organisms [6–8]. Recent advancements in cytogenetic techniques include fluorescence in situ hybridization (FISH) which can identify sex chromosomes through detection of sex-specific genes or loci using fluorescent DNA probes. The most popular gene used for this FISH analysis is the *dsx* and mab-3 related transcription factor 1 (*dmrt1*), a known master sex-determining gene in some animals. This FISH technique has led to the identification of sex determination systems of several animals such as the male heterogamety (XX/XY) for medaka fish *Oryzias latipes* [9] and female heterogamety (ZZ/ZW) for both African clawed frog *Xenopus laevis* [10] and domestic chicken *Gallus gallus domesticus* [11]. Recent approaches in identifying sex determination systems have taken advantage of the applications of high-throughput sequencing to identify the sex-linked markers. This approach has been applied to many animal species even without any prior robust cytogenetic information [12–14]. In fact, the XX/XY sex determination system in non-bilaterian animals was first inferred based on this method with the use of RAD-sequencing and SNP markers [15]. However, this approach requires genetic data from a high number of male and female individuals to differentiate sex-linked loci from the polymorphic loci in the autosomes [16]. In addition, due to its current limitations, bioinformatic and statistical tools are still being validated to accurately infer sex determination systems using these data [17]. A karyotypic analysis which offers direct observation of the chromosome structures and organization, as well as the loci of specific genes, might serve as important in-situ reference to explore its sex determination system. Although several cytogenetic data from non-bilaterians have been reported, all the species investigated are hermaphroditic, in which their karyotypes may provide no information on their sex chromosomes and thus to their GSD system [18–21]. Chromosome information from gonochoric non-bilaterian species would provide the evidence for identifying the GSD system for these animals.

Hence, in this study, we provide the GSD system for a gonochoric non-bilaterian *Goniopora djiboutiensis* based on cytogenetic data and FISH analysis. First, we karyotyped several metaphase cells to identify the presence of heteromorphic chromosome pairs, an indication of sex chromosomes. We then isolated the sperm-specific *dmrt* and identified its locus in their chromosomes. Here, we hypothesized that the *dmrt* locus is on one member of the heteromorphic chromosome pair, providing the evidence for the presence of the male chromosome and validate male heterogamety (XY) for *G*. *djiboutiensis*. We used *G*. *djiboutiensis* because of its commonality in many shallow coral reef ecosystems and established gonochorism [22, 23].

## Materials and methods

### Sample collection and chromosome preparation

**Fig 1:**
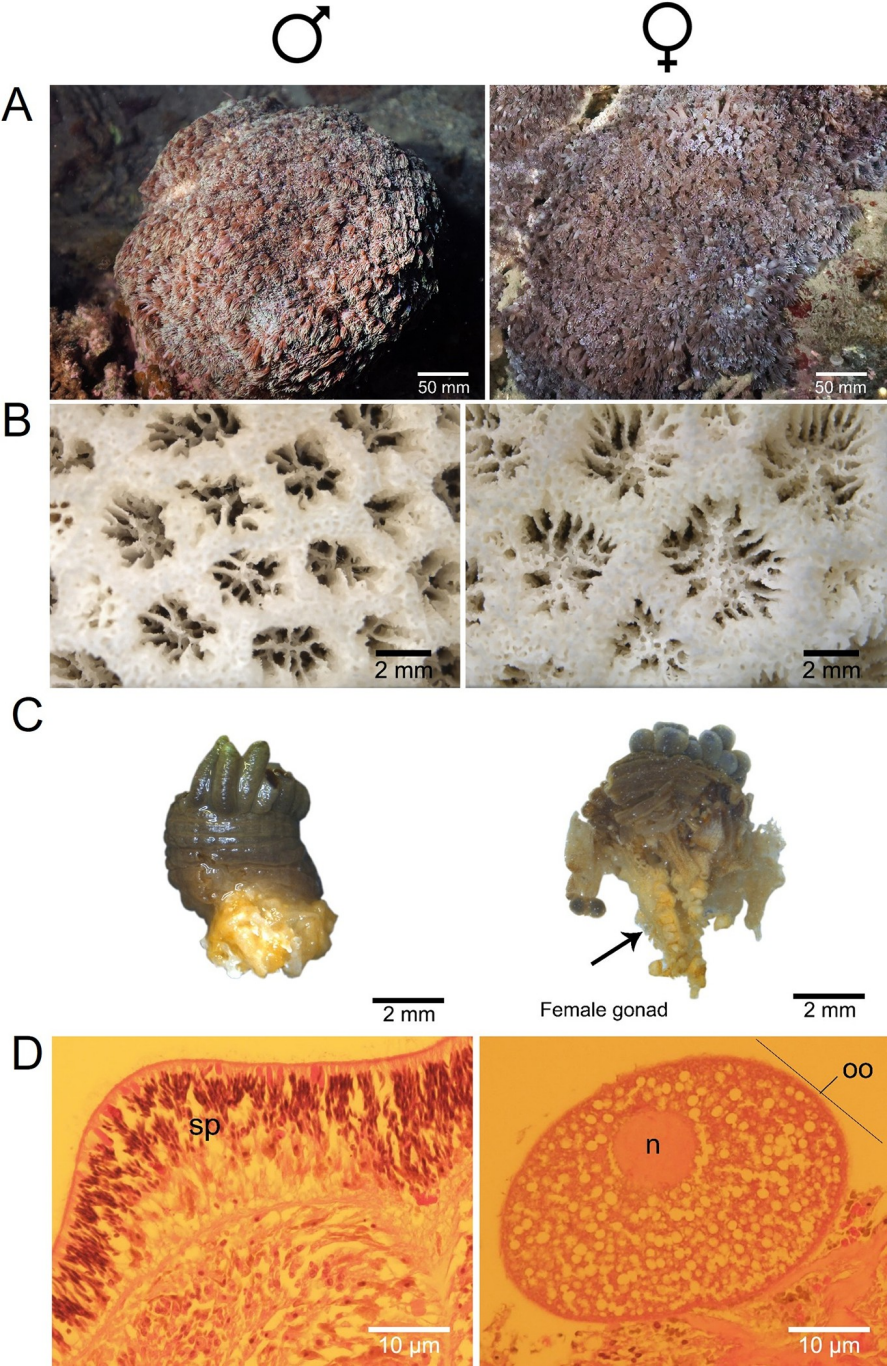
Morphological characteristics of male (left) and female (right) *Goniopora djiboutiensis*. (A) colony (B) corallites (C) polyps and (D) portion of their gonads. sp: spermaries, n: nucleus, oo: oocyte.

Eggs and sperms of the stony coral *G*. *djiboutiensis* were collected from separate colonies during its spawning in Otsuki, Kochi, Japan (32.777°N, 132.731°E). Both colonies spawned on the evening of August 29, 2021. Approximately 10 to 30 min after the male released its sperm, the female began releasing eggs. Comparisons of skeletal morphology of the two sexes from which the gametes were collected showed larger colony and wider corallite diameters in female than in male (Fig 1A and 1B). Aside from the spawned gametes, the sexes of the animal were confirmed by the presence of mature eggs and sperms in the gonads through microscopic observation (Fig 1C and 1D). A portion of the collected gametes were preserved in EtOH for DNA extraction, while the remaining gametes were combined and transferred in 0.2 μm filtered seawater to allow fertilization. The 12-hr-old embryos were then treated with 0.01% colchicine, followed by treatment with hypotonic solution (seawater: dH_2_0 = 1:1). Embryo samples were preserved in Carnoy’s fixative (absolute methanol: glacial acetic acid = 3:1) until further processing. Embryos were collected by centrifugation, and lipids were removed by 100% diethyl ether for 4–6 h. Cells were centrifuged at 2000 × g for 2 min and then resuspended in 0.5 mL of Carnoy’s fixative. Embryos were dissociated manually by rigorous pipetting. A cell suspension was dropped onto the slide and dried quickly by flame. For G-banding, dried chromosome slides were treated with 0.025% trypsin solution for 1 min, and then stained with 5% Giemsa solution diluted with 0.06 M phosphate buffer (pH 6.8) for 2 min before washing with dH_2_0.

Thirty-three metaphase spreads were observed which represent both the highly condensed and less-condensed chromosomes. Arm lengths of each chromosome were measured using the Drawid software [24]. Karyotyping was based primarily on chromosome length, as clearly revealed in DAPI staining. We used embryonic cells for chromosome analysis because they contain a substantial number of actively dividing cells, suitable to obtain high-quality metaphase spreads. In addition, adult tissues of corals are known to harbor endosymbiotic microalgae in which cells can contaminate with the target coral cells during the chromosome preparation. Collection of the samples was granted by permit no. 745 issued by Kochi prefectural government office.

### *Dmrt* amplicon preparation and RNA-seq

Genomic DNA from *G*. *djiboutiensis* sperm and eggs were extracted using Wizard Genomic DNA Purification Kit (Promega, USA) according to manufacturer’s protocol. Since no reference genome for *G*. *djiboutiensis* is available during the time of the experiment, we amplified the *dmrt* genes using its DM and DMA domain sequences, the known most conserved regions in protein structure of the cnidarian *dmrt* gene [25]. The conserved amino acid sequences for DM and DMA domains were obtained from http://pfam.xfam.org/, represented by a wide range of animal taxa. Sequences were then blasted (tblastn) against the NCBI database of transcriptome sequence assemblies from several cnidarian species. The mRNA transcripts were aligned and transformed to protein sequences, from which degenerate primers were designed. Forward degenerate primers were placed in DM domain, while reverse degenerate primers were placed in DMA domain. PCR were performed using Emerald PCR master mix (Takara, Japan) and sperm genomic DNA as a template. The PCR conditions were as follows: 30 cycles of 98°C for 20 s, 60°C for 30 s, and 72°C for 4 min. Target amplicon sizes based on the genome analysis of *Porites rus*, the closest related species to *G*. *djiboutiensis* with genome data, were excised from the gel and sequenced using the degenerate primers. From the sequence data, specific primers were then designed and used to re-amplify the gel-extracted DNA. Primer walking was conducted for amplicon sizes greater than 1 kbp. PCR products containing target amplicons were purified using AMPure XP beads (Pacific Biosciences, USA) before sequencing and probe preparation. We used the nearby internal primers for nested PCR to further assess the gene presence in the egg genome. S1 Table shows all the degenerate and specific primers used for each *dmrt* gene. Eight adult samples (4 males and 4 females) were collected for RNA extraction. The two males and two females were collected 3 months before spawning, during the gametogenesis stage as confirmed from the histological analysis. The other 2 males and 2 females were collected a day before spawning. Total RNA was extracted from following the method described by the manufacturer’s protocol for the Trizol reagent (Ambion, USA). Tissues from 0.5 g of coral fragments were solubilized with 2 ml of Trizol reagent and RNA was subsequently extracted using 250 μl isopropanol. A 10 μg extracted RNA was treated with 5 units of Recombinant DNase I (Takara, Japan). The crude total RNA was then purified using the standard ethanol precipitation. About 1 μg of total RNA were then sent for sequencing library preparation using the MGIEasy Library Prep Set (MGI, China) and the 150 bp paired-end reads were generated using the DNBSEQ-G400RS platform (MGI, China). Since no reference genome for *G*. *djiboutiensis* is available during the time of this study and de-novo transcriptome assembly was not possible due to low sequence coverage, the three *G*. *djiboutiensis dmrt* gene sequences were blasted against the *G*. *lobata* transcriptome assembly [26] to obtain the corresponding transcript sequences. Trimmed and quality filtered RNA-Seq reads were aligned to those reference transcripts using hisat2 [27] from which transcripts corresponding to the isolated *G*. *djiboutiensis dmrt* genes were assembled. Using the generated SAM files, consensus sequences of aligned reads were extracted using the Integrative Genomics Viewer (IGV) software [28]. Putative transcripts were then blasted using blastx against the UniProtKB/Swiss-Prot(swissprot) database. Protein alignment was based on the result of online BLASTP algorithm (https://blast.ncbi.nlm.nih.gov/) which gives an implicit alignment between the query and search database. The tree was then constructed using Grishin (protein) substitution model and FAST Minimum Evolution using the same online platform.

### Probe preparation and FISH

FISH probes were prepared from purified amplicons using the Random Primed DNA Labeling Kit (Roche, USA) according to the manufacturer’s protocol. The DNA was fluorescently labeled directly using cyanine-3-dUTP (Cy3-dUTP) (Enzo, USA) or indirectly using digoxigenin-dUTP (DIG-dUTP)/anti-Digoxigenin-FITC (Roche, USA) at 37°C for 15–18 h. Chromosome slides of *G*. *djiboutiensis* were denatured in 70% formamide in 2x Standard Saline Citrate (SSC) solution at 70°C for 2 min, and then serially submerged in ice-cold 70%, 90%, and 99% EtOH for 2 min each. About 1 μL each of the DNA probes of different labels were mixed with 20 μL hybridization solution and then probe mixtures were denatured at 80°C for 10 min. The probes were then gently placed onto the chromosomes denatured at 70°C, 2 min in 2x SSC, and slides were incubated at 37°C overnight with constant moisture to allow hybridization. Post hybridization washing was performed with 50% formamide in 2x SSC solution at 43°C for 20 min and slides were subsequently submerged twice in 2x SSC at 37°C for a total of 8 min. The slides were then incubated twice in 1X phosphate-buffered detergent (PBD) at 25°C for 5 min. Hybridized chromosome slides were then counterstained with DAPI-Vectashield (Vector Laboratories, USA) and viewed under an AxioImager A2 fluorescence microscope (Zeiss, Germany) equipped with an Axiocam MRm CCD camera (Zeiss, Germany). Primers for the preparation of the control histone H3 probe were based on related coral species *Favites pentagona* [29]. Images of suitable metaphase spreads from different embryos were captured using the AxioVision software (Zeiss, Germany).

## Results

### Karyotyping and chromosome structure

**Fig 2:**
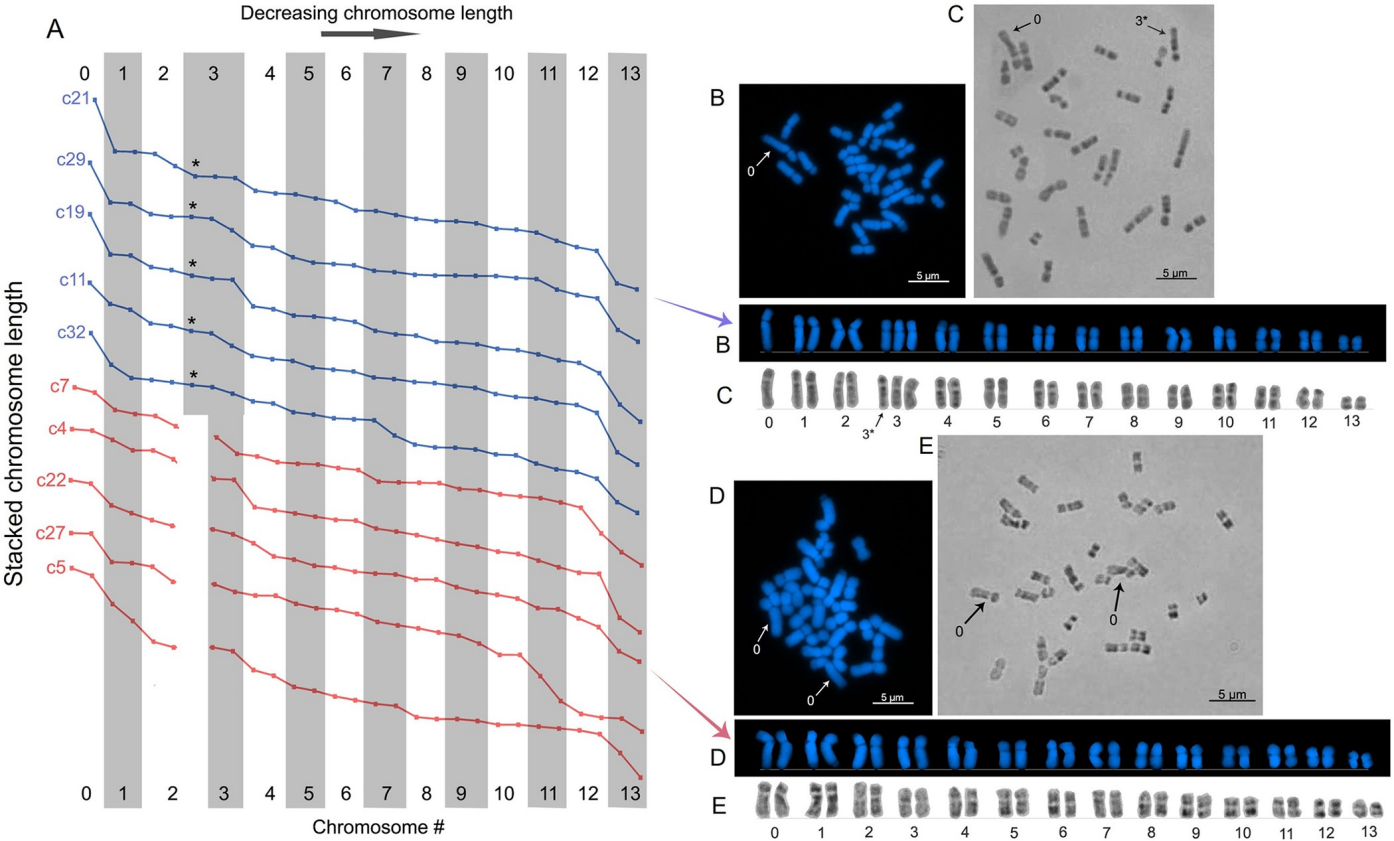
Karyotyping of *Goniopora djiboutiensis* metaphase cells. (A) Stacked chromosome length (μm) profile from 10 representative metaphase cells showing two types of karyotypes (blue and red trendline). (B) Karyotype c21 stained with DAPI showing the presence of heteromorphic pair composed of unpaired longest chromosome (chromosome 0) and additional chromosome 3 (3*). (C) Giemsa-stained metaphase cell of same karyotype showing the unique G-banding patterns of chromosome 3*. (D) Karyotype c22 stained with DAPI showing the pairing of the chromosome 0. (E) A metaphase cell of the same karyotype stained with Giemsa showing the absence of chromosome 3* with its distinctive G-banding patterns.

Chromosome lengths from 10 representative karyotypes with similar mitotic stages (composed of intermediately condensed chromosomes) showed the existence of two types of karyotypes (Fig 2A). The first karyotype (Fig 2A, blue trendline) has unpaired longest chromosome (tentatively named chromosome 0) based on its conspicuous length and its lower centromeric index compared to the next longest chromosome (Table 1). In addition, chromosome lengths alone indicate that this karyotype has three copies of chromosome 3 (Fig 2B). However, a careful inspection showed that one of the three chromosome 3 has a slightly different centromere position (Fig 2B and 2C) based on its lower centromeric index (Table 1). Further investigation using Giemsa staining also revealed that one of those 3 chromosomes has a different banding pattern by having 1 heterochromatic region in the short arm and 3 heterochromatic regions in the long arm (Fig 2C, 3* arrow). In contrast, the other two chromosome 3 have indistinguishable and lighter heterochromatin regions in the entire length of the chromosome. These observations suggest the presence of another unpaired chromosome (tentatively named chromosome 3*). These two unpaired chromosomes (chromosome 0 and 3*) in several cells are considered as the heteromorphic chromosome pairs, an indication of sex chromosomes. Although the other 23 non-representative karyotypes, which composed of long (less condensed) and short (highly condensed) chromosomes, showed inconspicuous size differences between each chromosome, identification of these heteromorphic pairs was still possible due to their distinct centromere locations and other chromosome features (i.e., chromosome width, stain intensity, secondary constriction).

**Table 1:** Average morphometrics of each chromosome pair from 33 metaphase cells of *Goniopora djiboutiensis*. Data are represented as mean ± SEM. Relative length is the ratio of the average length of the chromosome pair to its longest chromosome. Centromeric index is the ratio of short arm to chromosome length. The formula and classification are based on [30].

Chromosome #	Short arm length (μm)	Chromosome length (μm)	Relative length	Centromeric index X 100	Classification
0	1.83±0.53	5.96±1.53	0.99±0.02	30.78±3.88	Submedian
1	1.73±0.44	5.48±1.35	0.92±0.05	31.74±3.53	Submedian
2	1.75±0.47	5.13±1.18	0.86±0.07	34.24±4.63	Submedian
3*	1.73±0.4	4.92±1.15	0.84±0.07	35.3±2.96	Submedian
3	1.7±0.39	4.81±1.08	0.8±0.07	35.71±5.11	Submedian
4	1.49±0.55	4.44±1.03	0.74±0.05	33.67±9.15	Submedian
5	1.62±0.53	4.24±0.98	0.71±0.05	38.06±8.5	Submedian
6	1.62±0.48	4.11±0.97	0.69±0.04	39.94±8.5	Median
7	1.62±0.49	3.96±0.93	0.66±0.04	40.91±7.07	Median
8	1.61±0.41	3.83±0.9	0.64±0.05	42.29±7.02	Median
9	1.57±0.41	3.68±0.85	0.62±0.05	42.54±5.72	Median
10	1.57±0.39	3.53±0.77	0.59±0.05	44.3±4.77	Median
11	1.49±0.3	3.35±0.67	0.56±0.05	44.67±4.59	Median
12	1.38±0.28	3.09±0.55	0.52±0.06	44.77±4.84	Median
13	1.08±0.19	2.31±0.4	0.39±0.05	46.77±2.12	Median

*Chromosome is unpaired

The other karyotype (Fig 2A, red trendline) revealed that the chromosome 0 is paired (Fig 2D). This observation is supported by the conspicuous longer sizes of their first 3 chromosome pairs (chromosomes 0–2) than the rest of the chromosomes (Fig 2D and 2E), as compared with the karyotype with heteromorphic chromosome pairs in which one of those long chromosomes was missing (Fig 2B). In addition, these karyotypes are characterized by the absence of the unique chromosome 3*, as revealed in Giemsa staining (Fig 2E). The proportion of the two karyotypes observed in mitotic cells of *G*. *djiboutiensis* is 52% (17/33) for karyotypes with heteromorphic pair and 48% (16/33) for karyotypes with paired chromosome 0. The ratio of the two identified karyotypes is approximately 1:1, indicating the presence of a sex-specific karyotype. The average morphometrics of each identified chromosome pair from the 33 analysed metaphase cells showed that the chromosomes 0–5 including the chromosome 3* are all submedian types of chromosomes, while the chromosomes 6–13 are all median types (Table 1).

### Characterization of the *dmrt* genes

**Fig 3:**
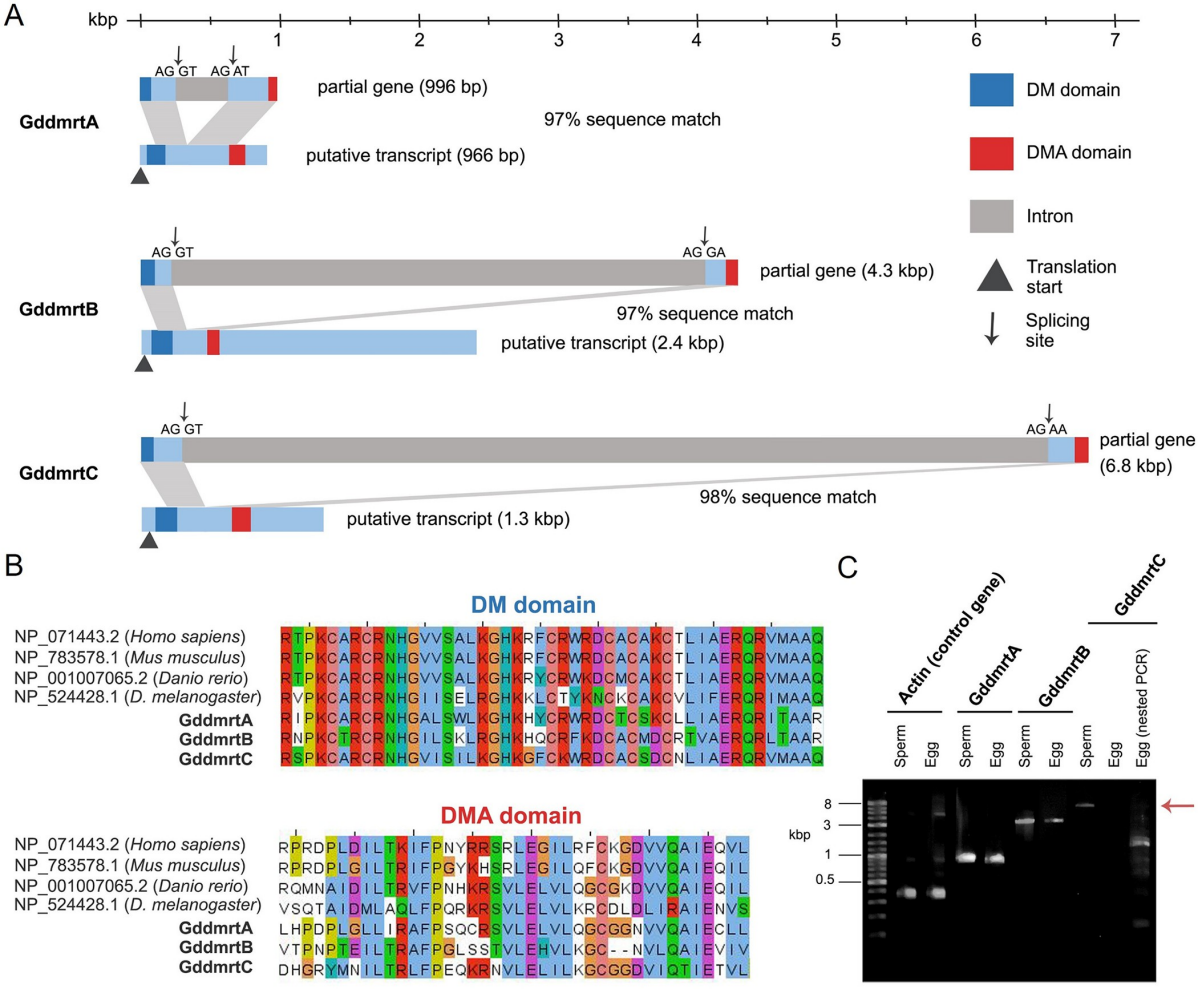
Sequence characterization of the 3 isolated *dmrt* genes (*GddmrtA*, *GddmrtB*, and *GddmrtC*) from *Goniopora djiboutiensis*. (A) Gene map showing the splicing sites and the locations of the DM and DMA domains. The corresponding transcript sequences were constructed based on the assembled RNA-seq reads. (B) Protein sequence alignment of the DM and DMA domains of the 3 *dmrt* genes along with sequences from model organisms. (C) Gel electrophoresis image of the amplified *dmrt* genes and the control *actin* gene from sperm and egg genomes. Expected band size for *GddmrtC* (red arrow).

Three *dmrt* genes were successfully isolated from the *G*. *djiboutiensis*. The genes were named *GddmrtA* (996 bp, NCBI accession no. LC704528), *GddmrtB* (4284 bp, NCBI accession no. LC704529), and *GddmrtC* (6762 bp, NCBI accession no. LC704530). As expected, all *dmrt* sequences contained the DM and the DMA domain, a common gene architecture of the *dmrt* (Fig 3A). Comparisons of the translated domains against that of wide range of animal groups showed the highly conserved DM domains, while DMA domains are less conserved (Fig 3B). Further inspection of the DM domains showed that among the three identified *G*. *djiboutiensis dmrt*, *GddmrtC* is most homologous to the *dmrt* of these model organisms.

PCR amplification with sperm and egg genomes showed that *GddmrtA* and *GddmrtB* are present in both gametes, while *GddmrtC* are present only in the sperm genome (Fig 3C, lane 8 and 9). To confirm any traces of the amplicon that may not have appeared visibly in the gel electrophoresis, we conducted nested PCR which showed persistent absence of the target *GddmrtC* band size (6.3 kbp) (Fig 3C, most right lane).

To determine the splicing sites of the sperm-specific *GddmrtC* gene, the sequence was blasted against the transcriptome assembly of hermaphroditic *Goniopora lobata* (http://www.comp.hkbu.edu.hk/~db/CoralTBase/index.php) [26]. The blast result (score: 542) outputs a single isoform of mRNA transcript (1.3 kbp) which contains the highly similar DM domain sequences and less similar DMA domain sequences. The RNA-Seq reads were then aligned to that transcript to assemble the corresponding *GddmrtC* transcript for *Goniopora djiboutiensis*. The *GddmrtC* gene map (Fig 3A) revealed the splicing sites in which the 5′ splicing site features the conventional GT/AG, a common splicing site for almost all eukaryotic genes [31, 32]. The upstream of the 5′ splicing site, which is an important recognition site for the U2 small nuclear ribonucleoprotein, consists of the putative CCGTTAG branch point, polypyrimidine motif CCTTTTT, and the consensus AG site in the 3′ end of the intron [33]. The 6.2 kbp intron region (37% GC content) contains no repetitive elements such as microsatellites and known transposable elements based on RepeatMasker analysis (http://www.repeatmasker.org/).

**Fig 4:**
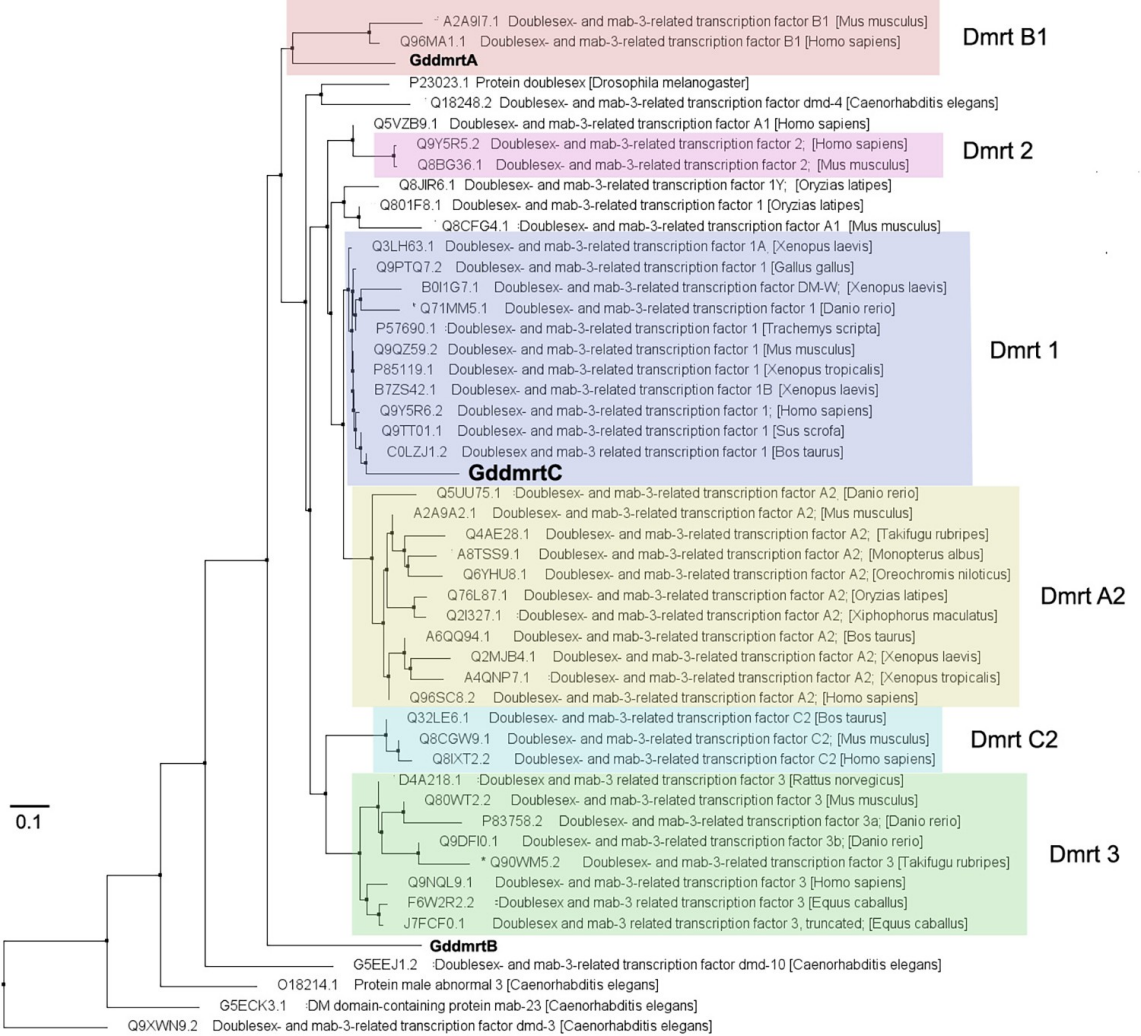
Distance-based tree of highly curated *dmrt* proteins and *Goniopora djiboutiensis dmrt* (*GddmrtA*, *GddmrtB*, *GddmrtC*) predicted protein sequences. Protein alignment was based on BLASTP pairwise alignments and Grishin (protein) substitution model. The tree was constructed using Fast Minimum Evolution method. *Dmrt* protein sequences were obtained from UniProtKB database.

Homology analysis of the translated coding regions of the three *dmrt* sequences revealed that the sperm-linked *GddmrtC* is most homologous to the doublesex- and mab-3-related transcription factor 1 (*dmrt1*) of the model organisms (Fig 4). Included in this cluster is the W chromosome-linked *dmrt* (DM-W) of African clawed frog *Xenopus laevis*. The protein sequence of the *GddmrtA*, on the other hand, is most homologous to *Dmrtb1* identified in mice and humans. The *GddmrtB* has the most divergent protein sequence, which appeared between most of the animal *dmrt* and *dmrt-dmd10* of *Caenorhabditis elegans*.

### *GddmrtC* gene loci

**Fig 5:**
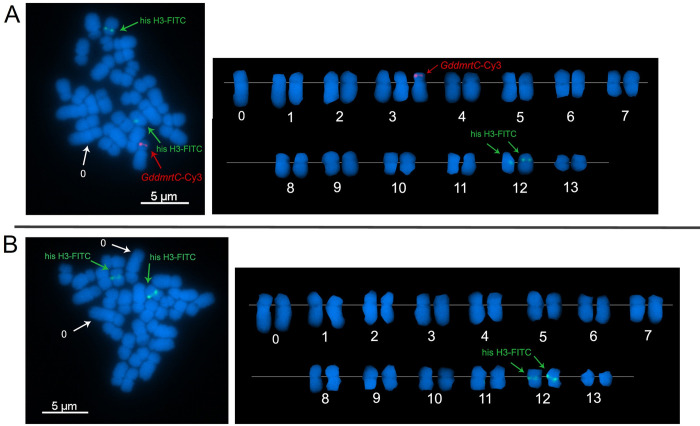
Locus identification of *GddmrtC* on the chromosomes of *Goniopora djiboutiensis*. (A) FISH image of metaphase spread with heteromorphic chromosome pair showing the locus of *GddmrtC* (red) and the control histone H3 gene (green). (B) FISH image of metaphase spread with paired chromosome 0 showing the locus only from the control histone H3 gene (green).

FISH analysis revealed that the locus of the sperm-specific *GddmrtC* gene (Fig 5A, red signal) was on the p-arm of a single chromosome. Karyotype showed that this chromosome is one of the chromosome 3 and appears to be the shorter chromosome of the heteromorphic pair (chromosome 3*) based on its unique centromere location. This revealed that the chromosome 3* contains the sperm-specific locus and possibly has the characteristics of the male chromosome Y. The locus of the control FISH probe (histone H3 gene), on the other hand, was detected on the chromosome pair of chromosome 12 (Fig 5A, green signal). This karyotype along with this FISH signal pattern was observed in 15 out of 32 (47%) metaphase spreads analysed by FISH, which is comparable to the 52% with heteromorphic chromosome pairs previously described. In addition, the unpaired longest chromosome in this FISH signal pattern resembles the karyotypes with heteromorphic chromosome pairs (Fig 2A, blue trendline).

The other 53% (17/32) of FISH images showed no locus for *GddmrtC* as shown by hybridization signal only from the control H3 probe (Fig 5B, green signal). Interestingly, these 17 cells also revealed karyotypes with pairing of the chromosome 0, which appeared to be the karyotypes previously described (Fig 2A, red trendline). The unpairing of the chromosome 0 in karyotypes that contained the putative chromosome Y and its pairing in karyotypes that do not contain the chromosome Y strongly suggests chromosome 0 as the female chromosome X. The proportion of the two FISH patterns observed is 47% and 53% (approximately 1:1 ratio), also indicating the presence of sex-specific karyotype.

To summarize, the result on karyotyping provided the evidence on the presence of heteromorphic chromosome pairs in almost half of the metaphase cells observed. Further, FISH analysis identified the locus of sperm-specific *dmrt* gene on the shorter chromosome of the heteromorphic pair. Combining these two results implies male heterogamety and suggests XX/XY sex determination system for *G*. *djiboutiensis*.

## Discussion

Heteromorphic chromosome pairs have already been observed in the karyotypes of non-bilaterians, particularly among stony corals [29, 34–36]. First attempts to identify these heteromorphic chromosome pairs as sex chromosomes were conducted in the chromosomes of *Acropora solitaryensis* [34] and *Acropora pruinosa* [36] using sperm DNA as the FISH probe. Although results showed intense hybridization signal on one member of the heteromorphic pair, this did not provide clear evidence whether the hybridized chromosomal region is composed of sperm-specific gene sequences.

In chromosomes of story coral *Favites pentagona*, a FISH probe from 18S ribosomal DNA (rDNA) sequences also showed intense hybridization signal on a single chromosome [29]. Studies have shown that repetitive 18S rDNA sequences, along with 28S and 5.8S, are part of the Nucleolar Organizing Region (NOR), known to reside in the sex chromosomes of some animals [37, 38]. In *Drosophila melanogaster*, this NOR in the sex chromosomes functions in the pairing of X and Y chromosomes during the metaphase I stage of meiosis [39]. The findings from *F*. *pentagona*, may infer that the 18S rDNA sequence can be used as FISH marker to identify the sex chromosomes. However, the 18S rDNA sequences are not exclusively located in the sex chromosomes, as it is also known to reside in the autosomes [40–42]. A better method is to use sex-specific genes as FISH probes to identify either the male or female sex chromosomes. Our study developed for the first time a FISH probe from sperm-specific *dmrt* gene to identify the male sex chromosomes in non-bilaterians.

Two of the isolated *dmrt* (*GddmrtA* and *GddmrtB*) are found to be non-sex specific. The *GddmrtA* is most homologous to *Dmrtb1*, which is autosomal in humans and plays a role in the entry of spermatogonia into meiosis [43]. *GddmrtB*, on the other hand, appears to be related to *Caenorhabditis elegans dmrt-dmd10* which functions in promoting neural signal of sensory receptor activation [44], a role not related to sex determination or gamete development. The autosomal characteristics of the *Dmrtb1* and the functional role of the *dmrt-dmb10* may support the non-sex specificity of the *GddmrtA* and *GddmrtB*, although there is greater possibility that chromosomal locations of certain genes might vary across different species. In contrast, the sperm-specific *GddmrtC* was most homologous to the *dmrt1*, in which experimental evidence has shown its involvement in male sex determination and differentiation by controlling the male gonad development [45–47]. In birds, the *dmrt1* gene is also linked to male Z-chromosome and knocking down the gene in males leads to transformation of the developing male gonads to female gonads [11]. The nucleotide sequence of the *GddmrtC* showed its highest similarity to the *dmrt* of *Acropora millepora* (*AmDM1*). Expression study of *AmDM1* showed that it undergoes alternative splicing that produces a transcript having both the *dmrt* domains (DM and DMA) and an alternative transcript having the DMA domain only [48]. The alternative transcript with the DMA domain only seems more involved in sex determination based on its higher expression during late embryonic stages when sex-specific gonad germ cells start to develop [48]. These studies on male-specificity and homology of the *GddmrtC* to *dmrt1* highly suggest its role as the master-sex determining gene in *G*. *djiboutiensis* and verify its potential use as chromosomal marker to identify the male sex chromosomes. It is important to note the possibility of existence of other sex-linked *dmrt* genes and their alternative spliced transcripts in *G*. *djiboutiensis* because the reference genomes and transcripts used in this study are from other species.

We found a single locus for the sperm-specific *GddmrtC* and that locus is located on the shorter chromosome of the heteromorphic chromosome pairs. These observations led to identification of the putative Y chromosome on *G*. *djiboutiensis*. *Dmrt* has also been reported to be sex chromosome-linked in other higher animals. For instance, DM-containing gene DMY is Y chromosome-linked in medaka fish *Oryzias latipes* (XX/XY system) [9], female-specific DM-W linked to W chromosomes of African clawed frog *Xenopus laevis* (ZZ/ZW system) [10], linkage of *dmrt1* in Z chromosome of domestic chicken *Gallus gallus domesticus* (ZZ/ZW system) [11], and linkage of *iDMY* in Y chromosome of Eastern spiny lobster *Sagmariasus verreauxi* [49]. Among the non-bilaterians, a study showed that in *Hydra*, the *dmrt* locus is on their homomorphic chromosome pair [50], but whether the pair is an autosome or sex chromosomes remains unknown. In case that this chromosome pair functions as their sex chromosome implies that its mechanism of sex determination may not be influenced by the single *dmrt* gene in the sex chromosome but rather mediated by dosage compensation or locus inactivation. There is limited information on the role of the number and action of the *dmrt* locus on the sex determination of non-bilaterians. The most recognized study on mechanism on sex determination among non-bilaterians is on *Hydra*, showing that its sex was determined by the presence of specific germline stem cells [51]. In that study, male polyps were found to originate from sperm-restricted stem cells, while female polyps originate from egg-restricted stem cells. Despite this discovery, the role of sex-determining genes and sex chromosomes in the formation of these sex-specific germ line cells has not been investigated yet in non-bilaterians. Our discovery of the Y chromosome-linked *dmrt* gene in *G*. *djiboutienesis* requires further investigation of its potential role as the master sex-determining gene in non-bilaterians. Likewise, future studies must also consider the possible influence of ESD on the role of these sex chromosomes and its associated *dmrt* genes.

The locus of the sperm-specific *dmrt* on the shorter chromosome of heteromorphic pair in the half of the population of cells analysed indicates male heterogamety and suggests XX/XY sex determination system for *G*. *djiboutiensis*. This is the first report on the cytogenetic identification of sex determination system using the locus of sex-specific gene in non-bilaterians. This method circumvents the problems associated with identifying the sex chromosomes based on traditional chromosome staining such as G-banding. Our findings therefore support the XX/XY sex determination system for gonochoric cnidarian, initially proposed based on genomic markers from the population of *Corallium rubrum* (Order Anthozoa) [15]. However, in *C*. *rubrum*, none of its *dmrt* analogs was found to be sex-specific or sex chromosome-specific. This is in contrast with our findings showing the linkage of one *dmrt* gene in the Y chromosome of *G*. *djiboutiensis*. Considering no *dmrt* is sex-linked in gonochoric *C*. *rubrum*, we speculate that the key genes involved in sex determination vary across different taxa of non-bilaterians.

The possible XX/XY sex determination system of cnidarian, as represented by precious coral *C*. *rubrum* and stony coral *G*. *djiboutiensis*, is similar with that of the mammals. However, cytogenetic study of other non-bilaterians such as in *Hydra* (Hydrozoan) showed no heteromorphic pairs, and its sex chromosomes might be homomorphic [21, 50]. Between the non-bilaterians and mammals are other various modes of sex determination system. These are male heterogamety (XX/XY) also in many insects; female heterogamety (ZZ/ZW) in birds, reptiles and Lepidoptera insects; homomorphic sex chromosomes in some reptiles; and haplodiploidy in some arthropods [1]. These convoluted patterns of sex determination system support the consensus understanding that sex chromosomes evolve independently across different lineages of animals. The evolutionary convergence of male heterogamety between highly distant animals is not surprising, as the XX/XY sex determination system is also manifested by many dioecious plants. It is widely proposed that the evolutionary process that results in this diversification of the sex determination systems involves the degeneration of the chromosome that acquired a sex-determining function. This degeneration is caused by the suppression of the non-recombining parts of the sex chromosome, which ensures that the advantageous alleles for a particular sex are linked and always coinherited [52, 53]. These chromosome events appeared to be continuous and reoccur frequently across different taxa, creating sex chromosome divergence and heteromorphy [1]. However, the time and the evolutionary pressure that drives sex chromosome evolution in animals is poorly understood. Estimates based on genomic data of the avian and gecko sex chromosomes revealed that the Z and W sex chromosomes started to differentiate at least 140 million-120 million years ago, before the split of most basal extant lineages [54]. In the case of male heterogamety, the time when the X and Y started to differentiate in any animal taxa has not been investigated, probably due to rare synapomorphy between large animal lineages. Within non-bilaterians, differentiated sex chromosomes was observed for anthozoans [15, 34, 36] and homomorphic sex chromosomes for hydrozoans [21, 50]. Because the phylogeny of the two taxa has not been established yet, it is difficult to confirm whether heteromorphic chromosomes evolved from homomorphic chromosomes in non-bilaterians. The other closely related invertebrate to non-bilaterians with known sex determination system is the *Caenorhabditis elegans* (X0), in which sex determination is not according to sex-limiting chromosomes but based on the counting mechanism of the X chromosome doses relative to the autosomes [55]. However, whether the sex determination in non-bilaterians depends on dosage composition of X rather than the role of the sex-determining gene in Y needs further investigation. Evaluating the sex determination systems from a wide range of animal taxa, either through cytogenetics or genomic analysis, would provide a better understanding in the patterns of lineage-specific evolution of sex chromosomes and GSD system in animals.

## Supporting information

S1 Table Primers used in this study. (DOCX)

S1 Fig Original and unadjusted photos of gels used in Fig 3C. (PDF)

S1 Raw images (PDF)
