# Supplementary material for: Cytogenetic evidence and dmrt linkage indicate male heterogamety in a non-bilaterian animal
Source: PLoS One. 2023 May 18;18(5):e0285851. doi: 10.1371/journal.pone.0285851 (PMC10194864; doi:10.1371/journal.pone.0285851)
Supplement: S1 Table — (DOCX) [file pone.0285851.s001.docx]

# Supporting information

**Table S1. Primers used in this study.** Highlighted in yellow are the degenerate primers. Highlighted in green are the internal specific primers used for nested PCR.

| **Gene** | **Primer name** | **Direction** | **Sequence (5'-3')** |
| --- | --- | --- | --- |
| GddmrtA | Coral_DM_F2 | F | AAGGGRCAYAAACACTACTGY |
|  | Coral_DMA_R2 | R | AGRACRCTNCGGCAYTGACT |
|  | Coral_dmrt_gF1 | F | GCTGGAGAGACTGTACCTGCT |
|  | Coral_dmrt_gR3 | R | TGACTCGGAAACGCTCGGATGA |
| GddmrtB | Gonio_DM4.3kbp_F1 | F | GGNCAYAARCAYCARTGYCG |
|  | Gonio_DMA4.3kbp_R1 | R | TGCARCCYTTYARCACGTGYTCYA |
|  | Gonio_DM4.3kbp_sF1 | F | CGCGATCTAGTTTCTAATCAG |
|  | Gonio_DM4.3kbp_sR1 | R | CTGACCTTCGCGATGTTCGTCTCC |
|  | Gonio_DMA4.3kbp_sR1 | R | CACCATAAACATCCTGCGCTT |
|  | Gonio_DMA4.3kbp_sF1 | F | CCTGAGAGCACCGACAGTGACTA |
|  | Gonio_DM4.3kbp_sF3 | F | GGGCACAAGCACCAATGCCG |
|  | Gonio_DM4.3kbp_sF4 | F | ATACAGCGCGGAATGATAGTAGC |
|  | Gonio_DMA4.3kbp_sR4 | R | CGCTCAACTCCTGAAGAAAGACGT |
|  | Gonio_DMA4.3kbp_sR3 | R | TGCAGCCCTTCAGCACGTGCTCCA |
|  | Gonio_DMRT4.3_sF5 | F | GTACATCGGGTGAAGTTCTCC |
|  | Gonio_DMRT4.3_sR5 | R | CGTTCGATCTGGTGATGAACTG |
|  | Gonio_DMRT4.3_sF6 | F | GAGCTGTCGTCCTTCGATACGT |
|  | Gonio_DMRT4.3_sR6 | R | CGATGGCTAACTTGAGCAAGT |
| GddmrtC | Gonio_DM6.4kbp_F2 | F | CACAARNGRTTYTGCAAATGG |
|  | Gonio_DMA6.4kbp_R1 | R | CARNACGTTYCNYTTCTGCTC |
|  | Gonio_DM6.4kbp_sF2 | F | GAGCAAGGCCAACTAGCAAGA |
|  | Gonio_DM6.4kbp_sR2 | R | TCTTGCTAGTTGGCCTTGCTC |
|  | Gonio_DMA6.4kbp_sR1 | F | GGCCTTCAGACATTGCACAATA |
|  | Gonio_DMA6.4kbp_sF1 | F | AACGCGATGAAGAAGATTCGG |
|  | Gonio_DM6.4kbp_sF3 | F | CACAAGGGGTTCTGCAAATGG |
|  | Gonio_DM6.4kbp_sF4 | F | TACAGCTCAGGCATAGGTTGCATG |
|  | Gonio_DMA6.4kbp_sR4 | R | CCGAATCTTCTTCATCGCGTT |
|  | Gonio_DMA6.4kbp_sR3 | R | CAGCACGTTCCGCTTCTGCTC |
| actin | Coral_Actin_qF2 | F | CCATGCCATAATTCGTCTCGAC |
|  | Coral_Actin_qR2 | R | ACTGTGTTGGCGTACAGATCCT |
| histone H3 | H3-F | F | ATGGCTCGTACCAAGCAGACVGC |
|  | H3-R | R | ATATCCTTRGGCATRATRGTGAC |
