# Supplementary material for: Cytogenetic evidence and dmrt linkage indicate male heterogamety in a non-bilaterian animal
Source: PLoS One. 2023 May 18;18(5):e0285851. doi: 10.1371/journal.pone.0285851 (PMC10194864; doi:10.1371/journal.pone.0285851)
Supplement: S1 Raw images — (PDF) [file pone.0285851.s003.pdf]

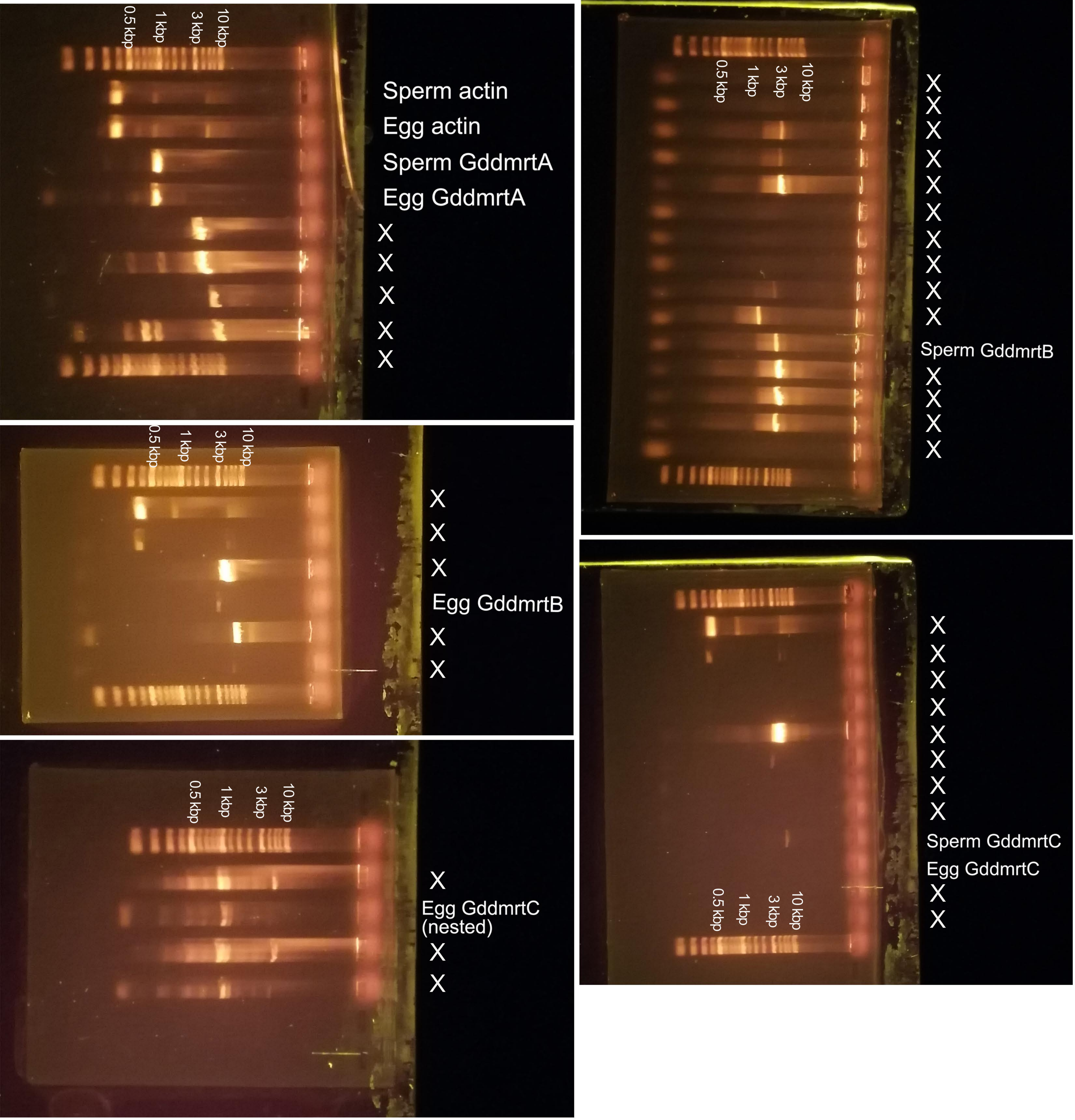

S1. Original and unadjusted photos of gels used in Fig 3C. Photos were taken using a smartphone camera. Lanes not included in the figure are marked with X.
